# Investigation of the effects of in ovo taurine injection on hatching characteristics and stress reduction potential

**DOI:** 10.1002/vms3.1387

**Published:** 2024-02-20

**Authors:** Yasin Baykalir, Ulku Gulcihan Simsek, İbrahim Seker, Abdurrahman Koseman, Burcu Gul, Mehmet Eroglu, Seda Iflazoglu Mutlu, Sezgin Kocyigit, Mehmet Karaca, Pelin Demir

**Affiliations:** ^1^ Department of Biostatistics, Faculty of Veterinary Medicine Balikesir University Balikesir Turkey; ^2^ Department of Animal Science, Faculty of Veterinary Medicine Firat University Elazig Turkey; ^3^ Akcadag Vocational School Turgut Ozal University Malatya Turkey; ^4^ Department of Nursing, Faculty of Health Sciences Firat University Elazig Turkey; ^5^ Agriculture and Rural Development Support Institution Elazig Turkey; ^6^ Department of Animal Nutrition and Nutritional Diseases, Faculty of Veterinary Medicine Firat University Elazig Turkey; ^7^ The Ministry of Agriculture and Forestry Elazig Turkey; ^8^ Department of Food Hygiene and Technology, Faculty of Veterinary Medicine Firat University Elazig Turkey

**Keywords:** hatching, HSP70, in ovo, stress, taurine

## Abstract

**Background:**

In ovo application is the process of administering some nutrients or components into the egg. The main purpose of this application is to ensure that some nutrients are provided to chicks with a short incubation period. Few studies were conducted with taurine in fertile eggs; especially, no observation of hatchability and chick quality has been found. In addition, taurine has an anti‐stress impact that fights oxidative factors.

**Objective:**

To assess the hatchability and chick quality after in ovo taurine administration. To determine the stress that may occur as a result of in ovo application and whether taurine has a stress‐reducing effect.

**Methods:**

A total of 1200 fertile eggs from a 34‐week‐old broiler breeder (Ross 308) flock were categorized into 4 groups with 75 eggs per replicate: control (uninjected), taurine group (0.30 mL dissolved taurine in distilled water), sham control (sterile distilled water) and perforation (eggs perforated and then waxed). On day 14 of incubation, an in ovo injection was administered to the albumen. Data on hatching parameters and hepatic HSP70 levels were obtained using relevant formulas and western blotting, respectively.

**Results:**

Control chicks exhibited higher hatchability than other groups, with the taurine group showing the lowest hatchability. The HSP70 levels were the highest in the perforation group compared to the control group. An increase of 21.37% in the taurine group and 83.45% in the sham control group was observed compared to the control group.

**Conclusions:**

The findings suggest that in ovo application may induce increased stress, whereas taurine may have positive effects in mitigating the stress caused by in ovo application.

## Graphical abstract

In ovo application causes stress. In ovo taurine administration showed a stress reduction effect the stress caused by in ovo application. Control chicks exhibited higher hatchability.

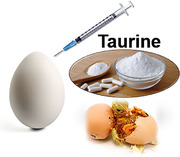

## 1 INTRODUCTION

Broiler production is crucial for meeting consumer demands for animal proteins (Ngongolo & Chota, 2021). Embryonic mortality is common in broilers during the early and late stages of hatching (Dey et al., 2019). Primary causes of mortality include issues with breeder flocks (such as health, nutrition and ageing) and failures in hatchery practices. Approximately 20% of chicks typically fail to hatch due to several factors, such as egg quality and chick weakness (Decuypere & Bruggeman, 2007), contributing to an increase in dead‐in‐shell chicks. Embryonic development in birds depends on available egg nutrients, and metabolic shifts during hatching depend on nutrient types. Utilization of glycogen stores, gluconeogenesis from amino acids and extensive muscle protein degradation towards the end of the incubation period can compromise chick development post‐hatching (Givisiez et al., 2020).

Research efforts to enhance broiler farming productivity have largely concentrated on hatchability, given that the embryonic stage (21 days) represents approximately 33%–38% of a bird's total life (Retes et al., 2018). Advances in egg micromanipulation techniques have opened new avenues for experimentation on unhatched birds. Various biological compounds (such as amino acids and their derivatives) can be administered through egg or embryo injections. However, in ovo injections influencing hatchability may unexpectedly harm rather than benefit, potentially ending an embryo's life prematurely. This effect is related to the injection's volume and concentration, not the substance used (Oliveira et al., 2023). Nevertheless, in ovo feeding remains a crucial technique (Givisiez et al., 2020; Nowaczewski et al., 2012).

Taurine, a colourless, water‐soluble, free amino acid weighing 125 Da, is not involved in protein biosynthesis and is abundant in the skeletal muscle and myocardium. Taurine is involved in numerous biological processes, including anti‐inflammation, anti‐oxidation, bile acid conjugation, membrane stability, osmoregulation, regulation of cellular calcium flux and immunomodulation (Miyazaki et al., 2023). Taurine exhibits protective effects in stress models, including endotoxin challenge, high stocking density and toxicity (Salze & Davis, 2015; Surai et al., 2020; Wang et al., 2009). Furthermore, taurine provides phospholipid stabilization in the sarcolemma and increases contraction elements’ sensitivity to calcium in the muscles, rendering the muscles to produce the necessary force and contraction and regulate the amount of intracellular calcium (Spriet & Whitfield, 2015). Therefore, taurine doses have varied significantly among the studies, generally in the range of 0.05%–0.50% of the diet. Taurine (0.3 mL) was administered in ovo into an air sack to determine the impact on muscle development in broiler chicks (Surai et al., 2020; Zielinska et al., 2012). Taurine biosynthesis is lower in infants than in adults because of limited cystathionase or cysteine oxidase enzyme activity. In ovo administration of taurine to broiler breeder eggs in the early embryonic period significantly increased the number of muscle cells and nuclei, muscle fibre diameter and proliferating cell nuclear antigen gene expression in the muscles (Zielinska et al., 2012). Some substrates and their combinations (carbohydrates, vitamin C and vitamin D3) have been tested via the in ovo route to determine how they affect the hatchability of birds (Baykalir et al., 2021; Salmanzadeh, 2012; Zhu et al., 2019). However, in one study, the in ovo administration of corticosteroids on embryonic day 7 resulted in a 35% decline in hatchability (Heiblum et al., 2001).

Heat shock proteins (HSPs), known as stress proteins, are present in all cells in all life forms but are expressed at high levels in cells exposed to high or low temperatures or other stressors (Figueiredo et al., 2007). Importantly, HSPs protect organisms against stress (Almalki et al., 2021) and prevent protein misfolding under stressful conditions. HSP70 is the most activated protein under any stress (Patir & Upadhyay, 2010). The role of HSP70 under heat and environmental stress has been extensively studied in broiler chickens (Hu et al., 2021) and laying hens (Baykalir & Simsek, 2018). However, there is no information on how HSP70 is influenced by in ovo administration in newly hatched chicks.

This study aimed to investigate the impact of in ovo taurine application on stress responses during incubation and determine whether taurine supplementation could alleviate stress‐induced effects, ultimately contributing to improved hatching performance.

## 2 MATERIALS AND METHODS

The use of animals and experimental procedures was conducted according to the guidelines of the Institutional Animal Experiments Local Ethics Committee of Inonu University, Malatya (protocol number: 2019/A‐46).

### 2.1 In ovo administration procedure and incubation management

Eggs from a 34‐week‐old commercial flock with an average weight of 57 g (55–60 g) were stored at 18°C and 75%–80% relative humidity for 3 days. Before the in ovo injection, all eggs were candled for fertility control, and 1200 fertile eggs were examined in 4 replicates. The eggs were sanitized on the commercial farm according to their procedure. The eggs were divided into 4 groups of 75 eggs per replicate: (1) control (without injection); (2) taurine group (0.3 mL taurine dissolved in distilled water); (3) sham control (sterile distilled water); (4) perforations (all eggs were perforated and then waxed). A stock solution for injection was prepared by dissolving high‐purity (≥99%) taurine at 4.32 mg/mL in autoclaved distilled water (Zielinska et al., 2012). All the treatment solutions were freshly prepared. All injection procedures were completed in the laboratory at room temperature, as previously described (Zhu et al., 2019). Under sterile conditions, taurine was injected with a 1 mL disposable syringe and a sterile disposable needle (diameter: 0.80 mm with a length of 38 mm (21G × 1.5). The injection time was at the embryonic age of 14 years, and the injection site was the egg albumen. At the end of the procedure, paraffin wax was used to cover the injection site. Before setting, the potassium permanganate and formaldehyde mixture was evaporated into an incubator for disinfection (40% formalin and 20 g potassium permanganate per cubic meter). The eggs were kept in the all‐in‐one cabinet‐type incubator with a capacity of 2400 eggs for 21 days. On incubation day 0, the incubator temperature was set to 37.8 ± 0.1°C and decreased to 37.1 ± 0.1°C on incubation day 18. The relative humidity was 65%. At the end of day 19, turning was stopped, and the humidity was adjusted to 72%. The number of turns was determined using an automated control device integrated into the incubator (24 turns). Fans were used to circulate air to maintain the temperature.

### 2.2 Assessment of hatching parameters

Hatched chicks from each group were weighed and recorded. Chick quality was determined by visual examination based on the activity of chicks, dryness, lively appearance of eyes, good leg posture and appearance of the umbilical region. Eggs that did not hatch post‐incubation were broken for macroscopic inspection to determine embryonic mortality. Mortality from day 15 until hatching was considered late embryonic mortality. Other hatching parameters and mortality rates were calculated using the following formulae Yousaf et al. (2017):

$$\mathrm{Hatchability}\;\mathrm{of}\;\mathrm{fertile}\;\mathrm{eggs}\;\left(\%\right)\;=\;\frac{\mathrm{number}\;\mathrm{of}\;\mathrm{hatched}\;\mathrm{chicks}\;}{\mathrm{number}\;\mathrm{of}\;\mathrm{fertile}\;\mathrm{eggs}}\;\times \;100$$

$$\begin{array}{ccc} & & \mathrm{Late}\;\mathrm{embryonic}\;\mathrm{mortality}\;\left(\%\right)\\ & & =\frac{\mathrm{number}\;\mathrm{of}\;\mathrm{late}\;\mathrm{embryonic}\;\mathrm{mortalities}\;}{\mathrm{total}\;\mathrm{number}\;\mathrm{of}\;\mathrm{fertile}\;\mathrm{eggs}}\;\times \;100 \end{array}$$

$$\mathrm{Dead}\;\mathrm{in}\;\mathrm{shell}\;\left(\%\right)\;=\;\frac{\mathrm{number}\;\mathrm{of}\;\mathrm{dead}\;\mathrm{in}\;\mathrm{shell}}{\mathrm{total}\;\mathrm{number}\;\mathrm{of}\;\mathrm{fertile}\;\mathrm{eggs}}\;\times \;100$$

$$\mathrm{Chick}\;\mathrm{yield}\;\left(\%\right)\;=\;\frac{\mathrm{weight}\;\mathrm{of}\;\mathrm{hatched}\;\mathrm{chicks}}{\mathrm{weight}\;\mathrm{of}\;\mathrm{set}\;\mathrm{eggs}}\;\times 100$$

$$\begin{array}{ccc} & & \mathrm{Moisture}\;\mathrm{loss}(\%)\\ & & =\frac{\mathrm{Weight}\;\mathrm{of}\;\mathrm{set}\;\mathrm{eggs}}{\mathrm{weight}\;\mathrm{of}\;\mathrm{set}\;\mathrm{eggs}-\mathrm{weight}\;\mathrm{of}\;\mathrm{eggs}\;\mathrm{at}\;\mathrm{incubation}\;\mathrm{day}\;18}\;\times \;100 \end{array}$$

### 2.3 Western blotting

To determine whether in ovo application would cause stress in chicks, hepatic HSP70 levels were examined via western blotting, as described by Luo et al. (2011). Briefly, 40 liver tissues (10 from each group) were homogenized in 1.50% KCl solution at a 1:10 (w:v) dilution rate. The total protein concentration was determined using the Lowry method (Lowry et al., 1951). Protein samples were loaded into the 12% SDS–PAGE resolving gel as 30 μg/30 μL final amount. Subsequently, the protein samples were transferred onto nitrocellulose membranes under semi‐dry conditions (Trans‐Blot Turbo system; Bio‐Rad). Rabbit monoclonal anti‐HSP70 antibody was used as the primary antibody, and goat anti‐rabbit IgG with horseradish peroxidase was used as the secondary antibody. For colorimetric band detection, the membrane was treated with the 3′3‐diaminobenzidine substrate. In addition, rabbit monoclonal β‐actin was used as a loading control for normalization using the same procedures. After the bands became visible, the relative optical density values (%) of the HSP70 bands were determined semi‐quantitatively using ImageJ software (NIH, CA, USA).

### 2.4 Statistical analysis

The experimental design was a randomized complete block with four treatments. All data were analysed using SPSS (version 20.0; IBM Corp.). The hatchability of fertile eggs and late embryonic mortality were subjected to a chi‐square test of independence. Chick yield, moisture loss and HSP70 data were analysed using a one‐way analysis of variance (ANOVA). To determine the differences between groups as a post hoc chi‐square test of independence, a 2 × 2 chi‐square test was applied. Tukey's post hoc test was used to determine differences between groups after the one‐way ANOVA test. Statistical significance was set at *p* ≤ 0.05. The following statistical model was used in this study:

$$\;Y_{ijkl}=\mu \;+\;C_{i}\;+\;T_{j}+S_{k}\;+P_{l}+e_{ijkl}$$

where *Y_ijkl_* is the performance of the examined characteristics, *C_i_* is the effect of the control, *T_j_* is the effect of in ovo taurin injection, *S_k_* is the effect of in ovo distilled water injection, *P_l_* is the effect of perforation on eggs only, *μ* indicates the effect of the overall mean and *e_ijkl_* refers to random effects.

## 3 RESULTS

In ovo administration resulted in significant differences in hatching parameters (Table 1). The differences in the hatchability of fertile eggs were statistically significant (*p *< 0.001). Any invasive treatment of eggs caused a significant decrease in the hatchability of fertile eggs, with the control group exhibiting the highest hatching rate. Embryonic mortality and dead‐in‐shell rates are presented in Table 2. Statistically significant differences were observed between the groups in terms of late embryonic mortality and dead‐in‐shell rates (*p *< 0.001). The taurine group showed the highest late embryonic mortality (27.57%), whereas the control group showed the lowest rate (7.33%). The dead‐in‐shell rates were the highest (13.33%) in the perforation group, followed by the control group (4.70%), taurine group (5.29%) and sham control group (1.67%).

**TABLE 1:** Group hatching parameters.

Hatchability of fertile eggs	Values (%)	df	*χ* ^2^	*p*‐Value
Control	89.70^a^	3	18.282	<0.001
Taurine (in ovo)	66.40^b^			
Sham control (in ovo)	76.10^b^			
Perforation	75.90^b^			

*Note*: Different superscripts (a,b) in the same column indicate differences between the groups.

Abbreviations: df, degree of freedom; *χ* ^2^, chi‐square value.

**TABLE 2:** Group mortalitiy rates.

Groups	Late embryonic mortalities (%)	Dead in shell (%)	df	*χ* ^2^	*p*‐Value
Control	7.33^a^	4.70^a^	3	17.414	<0.001
Taurine (in ovo)	27.57^b^	5.29^a^			
Sham control (in ovo)	20.33^b^	1.67^b^			
Perforation	9.24^b^	13.33^c^			

*Note*: different superscripts (a–c) in the same column indicate differences between groups.

Abbreviations: df, degree of freedom; *χ* ^2^, chi‐square values.

**FIGURE 1:**
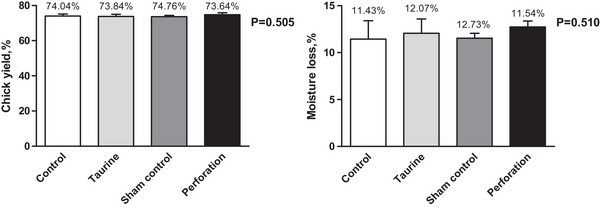
Chick yield and moisture loss of the groups.

**FIGURE 2:**
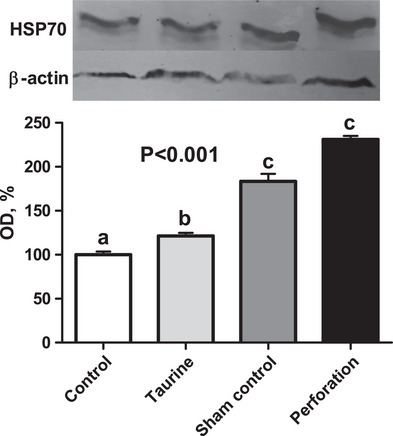
Hepatic HSP70 levels of the groups.

Chick yield and moisture loss are depicted in Figure 1, showing no statistically significant differences between the groups for either trait (*p *> 0.05). Hepatic HSP70 levels are shown in Figure 2. HSP70 levels were the highest in the perforation group, displaying an increase of 131.05% compared to the control group. The taurine group showed a 21.37% increase, and the sham control group demonstrated an 83.45% increase compared to the control group. These differences in HSP70 levels between the groups were statistically significant (*p *< 0.001).

## 4 DISCUSSION

Hatchability is an essential parameter in hatcheries, influenced by several factors. Fertility is primarily influenced by breed, nutrition, hen age and husbandry management (King'Ori, 2011). The presence of essential nutrients in eggs plays a vital role in the normal development of embryos and successful hatching. In commercial broiler farming, ensuring a good start for chicks in the hatchery is pivotal for maximizing profits, particularly considering the period (up to 48–72 h) until chicks have access to feed on the farm after hatching. Several studies have emphasized the importance of early nutrition (Kadam et al., 2013). To optimize hatchability, various nutrients have been introduced to eggs using the in ovo injection method, yielding diverse outcomes. For instance, glucose injection into the albumen (Ipek et al., 2004) and amino acid injection into the yolk sac on incubation day 7 (Ohta et al., 1999) showed no significant impact on hatchability. In contrast, the injection of glucose (Salmanzadeh, 2012) and corticosteroids (Patir & Upadhyay, 2010) into the albumen reduced the hatching percentage.

In this study, taurine was innovatively tested using an in ovo injection into the albumen. The taurine group demonstrated a decrease in hatchability compared with the control group. We created a perforation group beyond the sham control to observe the effects of in vitro administration on hatching parameters. Hatchability is strongly affected by in ovo administration. According to Ohta and Kidd (2001), the in ovo injection site and time substantially influence nutrient utilization via in ovo feeding. The optimal injection time is approximately 453 h (Salahi et al., 2011). In contrast, during incubation, nutrients pass from the yolk to the embryo through the yolk sac membrane and the surrounding vessels (Noble & Cocchi, 1990).

Certain amino acids have been tested in ovo, and hatchability was observed in broiler chicks. Cysteine, a sulphur‐containing amino acid similar to taurine, was detected in the air sac, with hatchability of 79.25% and 77.80% for uninjected eggs and sham controls, respectively (Ajayi et al., 2022). The hatchability of the cysteine group was 87.73%. Methionine, a sulphur‐containing amino acid, was injected into the air sac, similar to the procedure used for cysteine (Coskun et al., 2018). The hatchability of the uninjected eggs was 70.83%, sham control was 79.17% and methionine group was 77.08%. In our study, the hatchability of the uninjected, sham and taurine groups was 89.70%, 76.10% and 66.40%, respectively. These differences may be related to the site of injection. In contrast, taurine cannot pass through cells without sodium‐ or chloride‐dependent transport (Yang et al., 2009).

In this study, when these substances were added to the albumen, the hatching yield decreased, which may be associated with an allergic reaction caused by the injection into the albumen under the air sac, terminating the developing embryo's respiration (Salmanzadeh, 2012). The yolk sac may be more beneficial as the site of in ovo injection (Bhanja et al., 2008). Embryonic mortality was high, depending on several factors, between the 3rd and 5th days of incubation and again around the 19th day (Dey et al., 2019). Contamination may become a source of embryo mortality at any incubation stage, depending on infection severity. Manual in ovo manipulation may lead to the easy passing of microorganisms into the egg, causing contamination. Most embryos die during the last week because of adhesion and dehydration (Kalita et al., 2013). Mortality increased at the end of the incubation period. Moisture loss refers to the amount of water lost from the egg during incubation; this is vital for achieving good chicken quality and hatchability. Many factors, including humidity settings, damper positioning, variations in ventilation tolerance and atmospheric conditions, must be considered to achieve optimal moisture loss. The percentage of moisture loss can vary according to the age of the breeder flock, seasonal changes or egg size (Nasri et al., 2020). In single‐stage incubation, moisture loss is approximately 10.5%–12% in breeder flocks aged 31–50 weeks (Rahn & Ar, 1974). In this study, moisture loss was 11.43%–12.73% with single‐stage incubation and a breeder age of 34 weeks. Chick yield indicates the efficient utilization of stored egg resources to produce chicks, with a higher yield preferred in poultry hatchery practices. Herein, chick yield and moisture loss were not affected by in ovo administration.

HSPs are expressed in various tissues in most organisms in response to various stressors, including heat, chemicals and physiological stresses (Shehata et al., 2020). Many studies have been conducted on heat stress, investigating HSP alterations in poultry (Givisiez et al., 2001; Kang & Shim, 2021). However, there is no information on how in ovo treatment affects hepatic HSP70 levels in newly hatched chicks. A heat challenge during incubation caused an increase in hepatic HSP70 levels in chicks (Givisiez et al., 2001), with the lowest HSP70 level observed in the control group and an increase in HSP70 level observed in other groups. This may be caused by the impact of the in ovo application. However, the lowest HSP70 level was observed in the taurine group after the control treatment. Taurine contributes to antioxidant defence in different ways, including direct free radical scavenging and integrity maintenance of the mitochondrial electron transport chain under stress conditions (Surai et al., 2020). Therefore, the lower level of HSP70 in the taurine group compared to that in the sham and perforation groups was thought to be related to the antioxidant effect of taurine.

## 5 CONCLUSIONS

The study findings suggest that in ovo application may increase stress, and taurine could positively affect stress reduction. However, to better understand the effects of taurine on in ovo feeding, testing taurine with various nutrients at different injection sites and volumes is advisable. The sodium‐ or chloride‐dependent transport of taurine may pose a potential disadvantage in in ovo administration.

## AUTHOR CONTRIBUTIONS

*Conceptualization; data curation; investigation; project administration; supervision; writing – original draft; writing – review and editing*: Yasin Baykalir. *Formal analysis; investigation; validation*: Ulku Gulcihan Simsek. *Resources*: İbrahim Seker and Abdurrahman Koseman. *Formal analysis; methodology*: Burcu Gul. *Investigation; visualization*: Mehmet Eroğlu. *Formal analysis; visualization*: Seda Iflazoglu Mutlu. *Formal analysis*: Sezgin Kocyigit and Mehmet Karaca. *Software; writing – original draft; writing – review and editing*: Pelin Demir.

## CONFLICT OF INTEREST STATEMENT

The authors declare no conflicts of interest.

### ETHICS STATEMENT

The use of the animals and the experimental procedures were conducted under the guidelines of the Institutional Animal Experiments Local Ethics Committee of Inonu University, Malatya, Turkey.

### PEER REVIEW

The peer review history for this article is available at https://www.webofscience.com/api/gateway/wos/peer‐review/10.1002/vms3.1387.

## Data Availability

The data that support the findings of this study are available from the corresponding author upon reasonable request.
